# The Study of the Germination Dynamics of *Plasmopara viticola* Oospores Highlights the Presence of Phenotypic Synchrony With the Host

**DOI:** 10.3389/fmicb.2021.698586

**Published:** 2021-07-08

**Authors:** Giuliana Maddalena, Giuseppe Russo, Silvia L. Toffolatti

**Affiliations:** ^1^Dipartimento di Scienze Agrarie e Ambientali, Università degli Studi di Milano, Milan, Italy; ^2^Ordine dei Dottori Agronomi e Forestali di Milano, Milan, Italy

**Keywords:** dormancy, host adaptation, biotrophic pathogen, disease cycle, sexual reproduction

## Abstract

The plant disease onset is a complex event that occurs when the pathogen and the host encounter in a favorable environment. While the plant–pathogen interaction has been much investigated, little attention has been given to the phenological synchrony of the event, especially when both plant and pathogen overwinter, as in the case of grapevines and the downy mildew agent, the oomycete *Plasmopara viticola*. Oospores allow this obligate parasite to survive grapevine dormancy and, germinating, produce inoculum for primary infections. During overwintering, environmental factors influence the potential oospore germination. This study aimed at investigating the existence of synchrony between the pathogen and the host by identifying and quantifying the most important factors determining oospore maturation and germination and the relationship existing with grapevine phenology. Generalized linear models (GLM and GLMM) were used to analyze the germination dynamics of the oospores overwintered in controlled and field conditions and incubated in isothermal conditions, and oospore viability tests were carried out at different time points. Results showed that the most indicative parameter to describe the germination dynamics is the time spent by the oospores from the start of overwintering. The oospores overwintered in field showed phenological traits related to grapevine phenology not observed in controlled conditions. In particular, they completed the maturation period by the end of grapevine dormancy and germinated more rapidly at plant sprouting, when grapevine reaches susceptibility. Overall, the oospores proved to be able to modulate their behavior in close relationship with grapevine, showing a great adaptation to the host’s phenology.

## Introduction

Plants are not standalone entities; they interact with numerous organisms and microorganisms, in particular bacteria and fungi, that are located both in the endosphere and in the ectosphere (Vandenkoornhuyse et al., 2015), and can be beneficial or detrimental. In the case of fungal pathogens, in particular, the interaction with the plant occurs if phenological synchrony occurs between species (Ovaskainen et al., 2013). Concerning the interaction with fungal pathogens, research activity has mainly focused on the compatibility between species, to identify resistance traits to be employed for the control of the disease (Buonassisi et al., 2017; Sapkota et al., 2019; Sargolzaei et al., 2020). However, little attention has been given to the encounter between plant and pathogen, which determines whether and to what extent the parasite can find the host (Desprez-Loustau et al., 2010; Tack and Laine, 2014). Biotrophic fungal pathogens, in particular, are expected to have adapted to their host plants for phenological synchrony, to optimize the possibility of infection (Desprez-Loustau et al., 2010). Disrupting this synchrony could, indeed, be a way to prevent the disease onset.

*Plasmopara viticola*, is the biotrophic and obligate parasite causing downy mildew, one of the most economically impacting diseases of grapevine (Bois et al., 2017). *P. viticola* is a polycyclic pathogen that causes both primary and secondary infection cycles, leading to the complete loss of the crop in the absence of adequate disease control measures (Gessler et al., 2011; Toffolatti et al., 2018). In temperate climates, where grapevine undergoes dormancy, the pathogen differentiates oospores to survive the absence of the host. Even if alternative forms of survival have been recently postulated, thanks to the discovery of special *P. viticola* structures inside grapevine tissues (Fröbel and Zyprian, 2019), the sexual spores are considered to be the main survival structure of the pathogen. The oospores are large (25–50 μm diameter) thick-walled sexual spores that form, with different densities, within leaf tissues (Lehoczky, 1956; Si Ammour et al., 2020). They overwinter on the soil surface and germinate in the following grapevine-growing season, producing the inoculum for primary infections. During germination, the oospore differentiates, at the apex of the germ tube, a macrosporangium where the infection spores (the zoospores) are formed (Vercesi et al., 1999). Primary infections are an indirect consequence of the germination of the oospores: the inoculum is dispersed by wind and rain and zoospores infect susceptible grapevine tissues through stomata. Secondary infection cycles are caused by the production of asexual inoculum (sporangia differentiating new zoospores). Grapevine leaves are receptive to *P. viticola* for the whole vegetative season, whereas bunches terminate susceptibility with stomata closure (Kennelly et al., 2005): in the absence of open stomata, the zoospore cannot penetrate the berry tissues and start the infection process. The contribution of the inoculum produced by the oospores to downy mildew epidemics has been indirectly investigated through SSR (Simple Sequence Repeats) studies, where the presence of different genotypes in the pathogen population collected from infected leaves was linked to the infections caused by different oospore genotypes. Contrasting results, however, were achieved. In some cases, low population diversity of the pathogen in vineyards indicated that few genotypes originated secondary infections, suggesting the little importance of the inoculum produced by the oospores (Gobbin et al., 2005; Boso et al., 2019). On the other hand, the detection of new *P. viticola* genotypes during downy mildew epidemics and experimental infections with the oospores indicated that the oospores produce inoculum throughout the season (Kennelly et al., 2007).

Sexual reproduction in *P. viticola* is heterothallic (Wong et al., 2001; Scherer and Gisi, 2006) and the two mating types are called P1 and P2. Recent studies showed that the mating locus is heterozygous (MAT-a/MAT-b) in P1 and homozygous (MAT-a/MAT-a) in the P2 mating type (Dussert et al., 2020). The oospores are formed by conjugation of the sexual gametangia (oogonium and antheridium), where a single antheridial nucleus passes through a fertilization tube into the oosphere (the female gamete) (Burruano, 2000). The newly formed oospore goes through a maturation process during which the cytological and physiological processes necessary for survival and germination are completed (Widmer, 2010; Carisse, 2016). During this process, the antheridial and oogonial nuclei fuse, the multi-layered wall increases in thickness, a central vacuole (ooplast) is formed, and large lipid globules break into smaller ones (Vercesi et al., 1999). Many oospores do not complete maturation and die (Vercesi et al., 1999). Previous studies showed that the maturation period is completed over a variable interval of time: it can be really short for a small part of the field population, but it requires a long interval of time, up to 5 months, for most of the population (Ronzon-Tran Manh Sung and Clerjeau, 1988). The length of the maturation period for *P. viticola* oospores is established based on the time required by the oospores to reach the peak in the germination percentage at the optimal temperature of 20°C (Ronzon Tran Manh Sung and Clerjeau, 1989). Once mature, the oospores are able to germinate after a post-maturation (or after-ripening) period. After-ripening is a period of aging, where no discernible cytological changes occur, required by overwintering spores, such as oospores, to become germinable (Griffin, 1994). For *P. viticola* oospore, the post-maturation length is determined as the interval of time required by the oospores to produce the macrosporangium once incubated at 20°C (Ronzon Tran Manh Sung and Clerjeau, 1989; Burruano et al., 2006). As for maturation, the duration of the post-maturation period can also be variable, depending on the environmental conditions (Ronzon Tran Manh Sung and Clerjeau, 1989; Burruano et al., 2006).

Numerous studies investigated the influence of temperature (Ronzon Tran Manh Sung and Clerjeau, 1989; Burruano et al., 1990), water (Burruano et al., 1987; Rossi and Caffi, 2007), soil humidity (Burruano et al., 1992), and location (Galbiati and Longhin, 1984; Burruano et al., 1989) alone or in combination (Ronzon Tran Manh Sung and Clerjeau, 1989; Rossi et al., 2008; Vercesi et al., 2010) on oospore maturation and germination in natural and controlled overwintering conditions. To the best of our knowledge, the phenotypic synchrony existing between the pathogen and its host plant has not been investigated.

This study aimed at evaluating if oospore maturation and post-maturation show any connection with grapevine phenology. To this purpose, a combination of oospore germination and viability analyses, at different phenological stages of grapevine, was carried out on a population of *P. viticola* oospores divided into two subpopulations: the first one overwintered in field, and the second one in controlled conditions in the laboratory. These data were used to: (i) identify the most important variables affecting oospore germination, among temperature (average and cumulated), rainfall (cumulated and frequency of occurrence), and time from the start of overwintering; (ii) use the selected variables to estimate the duration of the maturation process [through generalized linear mixed model (GLMM) analysis] and post-maturation [through generalized linear model (GLM) analysis] of the oospores overwintered in different conditions (field and laboratory); and (iii) investigate if the oospore maturation and post-maturation variables show a relationship with the host plant phenology by comparing the results obtained in the two overwintering conditions. We hypothesized that synchrony with the host should occur in field- but not in laboratory-overwintered samples.

## Materials and Methods

### Vineyard

Experimental activities were carried out in a vineyard of 13-year-old plants of cultivar Corvina grafted onto Kober 5BB with pergola Veronese training system, located in the province of Verona (Northern-eastern Italy, Veneto region) at Montorio (MT vineyard). Phenological stages of grapevine were weekly recorded from the beginning of sprouting until bunch closure (end of bunch receptivity to *P. viticola*) by using the BBCH scale (Lorenz et al., 1994). Meteorological data (daily temperatures and rainfall) were collected from an *in situ* weather station and used to calculate monthly values of average temperature (T), sum of average temperature (SOT), total rainfall (R), cumulated rainfall (CR), and frequency of rainfall (FR) (Supplementary Table 1).

### Sample Preparation

Grapevine leaves showing downy mildew symptoms were sampled for four consecutive years at the end of October from a plot (consisting of three rows 60 m long) that was not treated with fungicides against *P. viticola*. Since sexual reproduction in infected leaf tissues is recorded from early summer onward (Burruano, 2000), oospores of different ages can coexist in the plants. Therefore, particular care was taken in collecting the younger leaves (Supplementary Figure 1A) that were formed and infected late in the season and should present a more uniform age interval. Leaf portions (∼1 cm^2^) containing oospores were cut with a razor blade at the microscope (Leitz Orthoplan) (Supplementary Figure 1A) and placed in nylon bags with 100-μm pores (Supplementary Figure 1B; Vercesi et al., 2010). Each nylon bag contained 20 leaf fragments, cut from different leaves, to achieve a good representation of the variability existing in vineyard. The nylon bags were divided into two groups, each consisting of three series (biological replicates) of 70 samples, and overwintered on the soil surface in vineyard (MT samples) or in controlled conditions in the laboratory (MTc samples; Supplementary Figure 1C). The three series of MT samples were randomly positioned below grapevine plants in three different rows of the vineyard. Adherence to the soil of the samples was maintained by using anti-hail nets (Supplementary Figure 1C). The three series of MTc samples were placed in three different incubators with 65% relative humidity and kept at 5°C on the surface of a sand substrate (Supplementary Figure 1D) constantly water-saturated, conditions that are highly favorable for the oospore overwintering (Vercesi et al., 2010).

### Germination Assays

Germination assays were carried out twice a week (every 3 and 4 days) for 35 weeks (Supplementary Figure 1E) at different phenological stages of grapevine, from start of overwintering (mid-November) until the end of bunch receptivity (mid-July). The number of days passed from the start of overwintering (DFO = days from the start of overwintering) was recorded for each germination assay. The oospore germination was estimated on the three biological replicates of individual MT and MTc samples (Supplementary Figure 2A) according to a water-agar protocol (Ronzon-Tran Manh Sung and Clerjeau, 1988; Tran Manh Sung et al., 1990; Vercesi et al., 2010). Briefly, leaf samples from each nylon bag were homogenized with a glass Potter tissue grinder (Thermo Fisher Scientific) in sterile, distilled water. The oospores were collected, after double filtering with 100-μm and 45-μm pore gauze, in a few milliliters of sterile distilled water (Supplementary Figure 2B) and inoculated on 1% water-agar (Agar Noble, Difco, Thermo Fisher Scientific) (Vercesi et al., 2010). The oospore suspension consisted of a heterogeneous combination of mature, immature, and dead structures. For each nylon bag, three agar plates (technical replicates) were inoculated with four 10-μl droplets of 100 oospores. A total number of 1200 oospores was therefore analyzed per nylon bag (Supplementary Figure 2C). The number of germinated oospores was checked at the stereomicroscope (Leica Wild M10) from 1 to 14 days after incubation (dai) at the optimal temperature of 20°C (Galbiati and Longhin, 1984; Rouzet and Jacquin, 2003; Supplementary Figure 2C). These data were used to calculate, for each germination assay, the average germination percentage (G) at 14 dai (Vercesi et al., 2010) and the cumulated number of germinated oospores from 1 to 14 dai (Gcum) (Supplementary Figure 3A). Isothermal conditions were chosen to estimate the effect of the two overwintering conditions by avoiding any interference of incubation conditions on the oospore germination. Cumulated values of G, which are used for modeling fungal spore germination (Eisensmith et al., 1985; Peleg and Normand, 2013), were used for the estimation and comparison of maturation and post-maturation parameters of MT and MTc samples as performed on other fungal species such as *Penicillium expansum* and *Aspergillus niger* (Gougouli and Koutsoumanis, 2012).

### Oospore Viability

Viability, defined as a percentage of live cells in a whole population (Kwolek-Mirek and Zadrag-Tecza, 2014), was assessed by trypan blue staining (Toffolatti et al., 2007) at year 4 on MTc oospores. The viability of the oospores overwintered in field conditions was not investigated in the present study, since previous data were reported on these conditions (Toffolatti et al., 2007). Part of the oospores used for the germination assays at 0, 60, 90, 120, and 180 DFO were placed in nylon bags with 45-μm pore size, immersed in a 40-ml solution containing 1 g/L trypan blue (glycerol 10 ml, lactic acid 10 ml, phenol 10 ml, and trypan blue 40 mg in 10 ml of water) and boiled for 2 min. The oospores were then cleared in 10 ml of 2.5 g/ml chloral hydrate-water solution for 4 h and observed under an optical microscope (Zeiss Primo Vert, 40× magnification). Reagents were purchased from Merck (Milano, Italy). Trypan blue is a dye, commonly used in mycology, for assessing cell vitality: cells with intact cell membrane, in fact, exclude the dye and do not change color, while dead cells get a blue color (Strober, 1997). The number of alive (not colored) and dead (blue-colored) oospores was counted on three replicates of 100 oospores for each sample, and viability of the oospores (VO) was calculated.

### Estimation of Maturation and Post-maturation Variables Through GLMM and GLM Models

The values of Gcum (GO) recorded at 14 dai in each germination assay of MT and MTc samples were cumulated over DFO (Supplementary Figure 3B). The length of the maturation period was determined as the DFO value at which the observed cumulative oospore germination reached 50% (germination peak) at the optimal temperature of 20°C (Ronzon-Tran Manh Sung and Clerjeau, 1988; Ronzon Tran Manh Sung and Clerjeau, 1989). A GLMM was used to estimate the GO values as a function of DFO and SOT, which were used as fixed and random slopes, respectively. The model coefficients were then used to compute the DFO_50_ values, assuming a median SOT. The upper limit (UL) and lower limit (LL) of DFO_50_ values were determined through bootstrap computation (250 replications) and used to compare MT and MTc samples (Badiru and Ijaduola, 2009). GLMM Probit model was fitted by glmer() function implemented in lme4 R 3.4.3 package, while bootstrap computations were performed by bootstrap function implemented in bootstrap R 3.4.3 package. The GLMM goodness of fit was obtained by observed vs. simulated linear regression in order to compute the pseudo-*R*^2^ (Piñeiro et al., 2008). The details of the modeling procedure are reported in Supplementary Appendix.

The length of the post-maturation period (T_50_) was estimated as the number of dai required by the oospores to reach 50% of Gcum in the individual germination assays carried out on MT and MTc samples at different DFO (Supplementary Figure 3A). Time to reach 50% germination is a parameter used to compare the effect of treatments on plant seeds (Farooq et al., 2005; Joosen et al., 2010) and fungal spores (Gottlieb, 1950). T_50_ was calculated through GLM (logit link) of Gcum values by using SPSS v. 27 software (IBM Milano, Italy). The goodness of fit of the logistic model was evaluated graphically and by the calculation of the pseudo-coefficient of determination (pseudo-*R*^2^) (Cox and Snell, 1989).

### Additional Statistical Analyses

Unless otherwise stated, statistical analyses were carried out by using SPSS v. 27 software. ANOVA and multiple comparison of the mean with REGW test were performed on transformed percentages ($\text{asin}\sqrt{(}\%/100))$ of germination (G) and viable oospores (VO) at different DFO intervals. Shapiro–Wilk and Levene’s tests were conducted prior to ANOVA. Principal component analysis (PCA) was carried out on monthly values of germination percentages (G), temperatures (T), sum of temperatures (SOT), total rainfall (R), cumulated rainfall (CR), and frequency of rainfall (FR) of MT samples between year 1 and 4 using the varimax rotation option. The existence of correlation between T_50_ and DFO values, reflecting different phenological stages of grapevine (BBCH scale), was estimated through linear regression of MT and MTc samples by GraphPad Prism 8 software. The slopes and elevations of linear regression lines of MT and MTc samples within a year were compared by GraphPad Prism 8 software with an analysis of covariance (ANCOVA) approach (Zar, 2010). Differences between the average values of T_50_ at different DFO intervals (0–30, 31–60, 61–90, 91–120, 121–150, 151–180, 181–210, and 211–240 days from the start of overwintering) were evaluated by ANOVA and multiple comparison of the mean values following REGWF test.

## Results

### Oospore Germination

**FIGURE 1:**
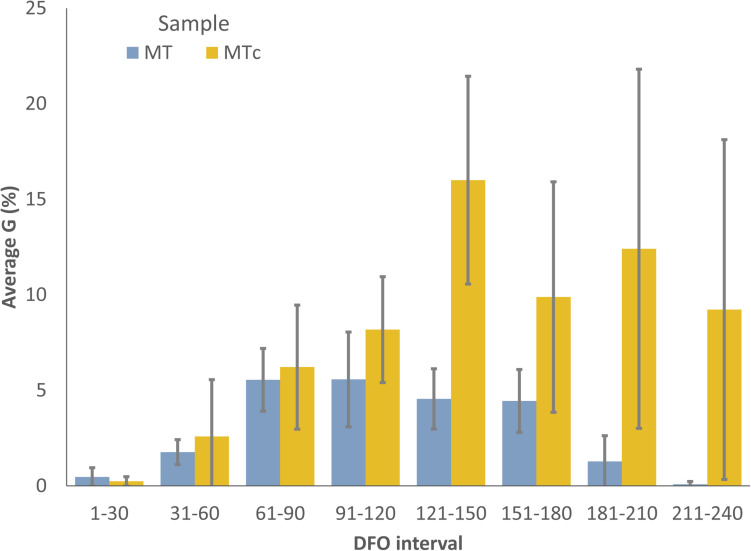
Average germination percentages of MT (blue) and MTc (yellow) samples at different DFO intervals. Bars represent 95% CI.

MT oospores showed a significant and progressive increase in the germination percentages during grapevine dormancy, going from 0.02–2.6% (1–31 DFO; BBCH = 0) to 3.4–9.5% at the end of dormancy (61–120 DFO; BBCH = 0) in years 1, 2, and 4 or end of dormancy-sprouting (121–180 DFO; BBCH = 0–13) in year 3 (Table 1). A decreasing trend immediately afterward led to complete absence of germination from the end of flowering (210 DFO; BBCH > 68) onward in year 3, while a few oospores also germinated at flowering and fruit development (211–240 DFO; BBCH > 60) in years 1, 2, and 4 (Table 1). The G values of MTc oospores significantly increased with the number of cold treatment days from 0 (G = 0.1–0.4%) to 121–150 (G = 12.2–17.6%) DFO, when the samples often reached the highest G values (Table 1). Between grapevine sprouting and flowering (151–210 DFO; BBCH = 1–69), G showed different behaviors: it fluctuated between 20 and 8%–10% in year 1, significantly decreased to values lower than 7% in years 2 and 3, and it stably remained approximately 18–21%, with no significant increase, in year 4. In any case, increasing the length of cold storage from 121 to 150 DFO onward did not result in any increment of the germination percentage of the oospores. On average, during the 4 years of analyses, the G values recorded by MT and MTc samples did not significantly differ (0.15 < *F* < 1.6; df = 1–22; *p* > 0.2) during grapevine dormancy (1–120 DFO; BBCH = 0) (Figure 1). The two sample series started diverging from the end of grapevine dormancy (121–150 DFO, BBCH = 0) onward (DFO 151–240; BBCH = 1–79): in these stages, MTc samples showed significantly higher (8.7 < *F* < 64.7; df = 1–22; *p* < 0.007) G values than MT samples (Figure 1). On the contrary, from sprouting onward (DFO > 151; BBCH > 1), G values of MT samples showed a progressive and significant decrease leading to a very sporadic oospore germination at flowering and fruit development (211–240 DFO; BBCH = 60–79) (Table 1).

**TABLE 1:** Phenological stage of grapevine (BBCH scale^*a*^) and average germination percentages of *P. viticola* oospores, which had been stored under field (MT) or controlled (MTc) conditions for 0–240 days (DFO = Days from Overwintering) and incubated at 20°C for 14 days and results of statistical analysis (ANOVA and multiple comparison of mean values^*b*^) between years 1 and 4 and indication of the phenological stages of grapevine^*a*^.

DFO	BBCH stage				MT				MTc			
			
					1	2	3	4	1	2	3	4
1–30	0	0	0	0	0.3a	0.3a	0.02a	1.2b	0.4a	0.1a	0.1a	0.4a
31–60	0	0	0	0	1.7b	1.0b	1.8b	2.6c	1.8ab	0.9a	0.6ab	7.0ab
61–90	0	0	0	0	7.0*e*	3.4*de*	4.8c	7.0e	5.4ab	5.1ab	4.3ab	10.1abc
91–120	0	0	0	0	4.6d	3.7e	4.5c	9.5f	9.9ab	6.9ab	8.0b	8.0abc
121–150	0	0	0	0	3.4c	2.9*cd*	6.5d	5.4d	19.5d	12.2b	14.7c	17.6*de*
151–180	1–9	1–13	1–13	1–19	3.4c	2.8c	6.8d	4.8d	10.7bc	5.1ab	6.2ab	17.6*cde*
181–210	11–61	14–57	15–65	53–69	3.5c	0.6ab	0.8a	0.2a	21.9d	4.6ab	4.2ab	19.0*de*
211–240	63–75	60–75	68–77	71–79	0.3a	0.02a	0a	0.03a	7.9ab	0.7a	7.0ab	21.2e
				*F*	152.1	121.2	153.2	263.0	11.9	5.4	7.7	7.1
				df	7–16	7–16	7–16	7–16	7–16	7–16	7–16	7–16
				*p*	<0.001	<0.001	<0.001	<0.001	<0.001	0.003	<0.001	0.001

*^*a*^0 = dormancy; 1–8 = sprouting; 11–19 = leaf development; 53–57 = inflorescences emerge; 60–68 = flowering; 71–79 = development of fruits. ^*b*^Different letters correspond to significant differences within a year for α = 0.05.*

### Identification of the Most Important Parameters Influencing the Oospore Germination in Field Conditions

**FIGURE 2:**
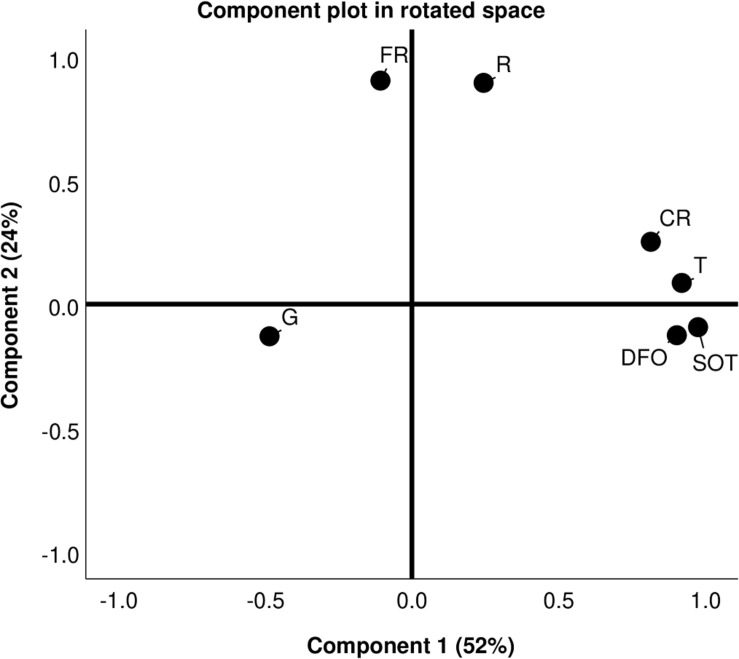
Principal component analysis graph showing the monthly values of germination percentages (G), temperatures (T), sum of temperatures (SOT), rainfall (R), cumulated rainfall (CR), and frequency of rainfall (FR) of MT vineyard between year 1 and 4.

The phenological stages of grapevine and the monthly values of T, SOT, R, CR, FR, and DFO are reported in Supplementary Table 1, while the monthly values of G are reported in Table 1. In general, T followed a decreasing trend from 8–10°C to 2°C in the late autumn–winter period (31–120 DFO), increased above 10°C at 151–180 DFO (grapevine leaf development stage, BBCH = 1–19), and reached the highest values in spring–summer reaching 20°C–27°C at 211-240 DFO (flowering and fruit development, BBCH = 60–79). SOT showed a progressive increase in time that followed an exponential growth between 120 and 240 DFO, a period going from the end of grapevine dormancy onward. The total rainfall amount per year ranged from 276 to 610 mm, as indicated by final CR values. Based on R and FR values, rain was less frequent and less intense in the winter period (61–150 DFO, BBCH = 0) and most frequent and intense at the beginning of overwintering (1–60 DFO, BBCH = 0) and at the start of the new vegetative season of grapevine (151–180 DFO, BBCH = 1–19). The results obtained with principal component analysis (Figure 2) showed that the total variance (76%) was mostly explained by the first component (accounting for 52% of the total variance) followed by the second component (accounting for the remaining 24% of variance). The first principal component strongly correlated with five variables (G,T,SOT,DFO, and CR) and showed an increasing trend with T, SOT, DFO, and CR. This suggests that these three variables vary together. However, the first principal components decreased with G values, indicating that increasing T, SOT, DFO, and CR were negatively correlated with the germination of the oospores. The second principal component grouped together R and FR, and separated G, SOT, and DFO from the other variables (Figure 2). As a consequence, the effects of SOT and DFO on G were more thoroughly investigated, as described in the following paragraphs.

### Estimation of the Parameters Associated With the Completion of Oospore Maturation

**FIGURE 3:**
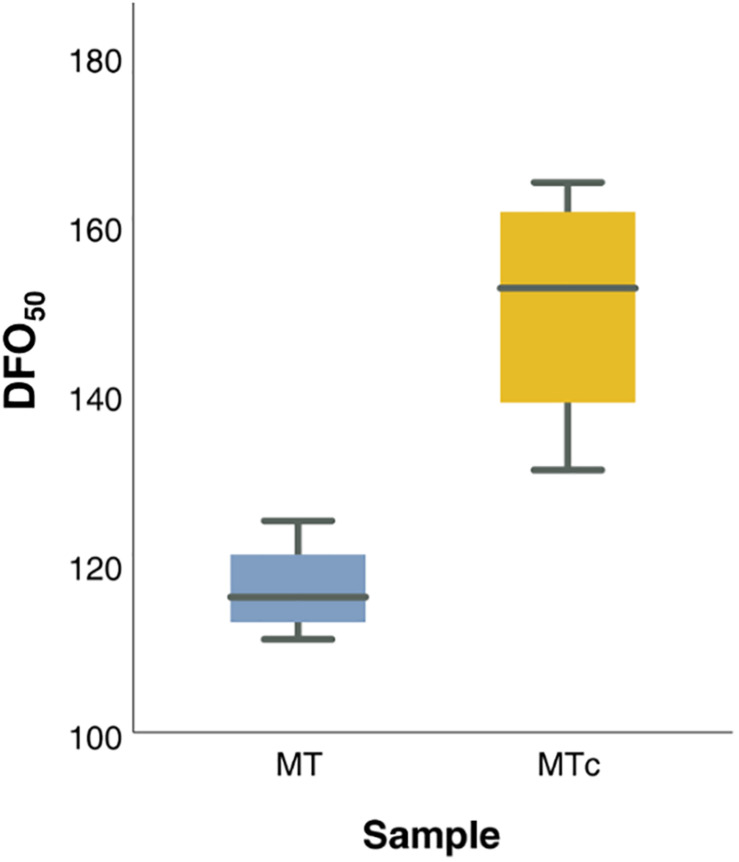
Box-plot distribution of DFO_50_ values of MT (blue) and MTc (yellow) samples during the 4 years of investigation.

**FIGURE 4:**
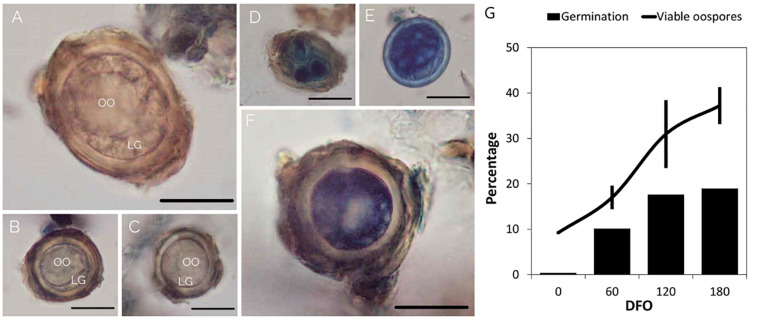
Results of viability assay. Pictures of living **(A–C)** and dead, blue-colored, oospores **(D–F)**. Bar = 25 μm. OO = ooplast; LG = lipid globules. **(G)** Percentages of germination (bar) and viable oospores (line) of MTc samples. Bars in the line graph represent standard deviation.

The maturation process was considered complete when the oospores reached the peaks in germination (Ronzon Tran Manh Sung and Clerjeau, 1989) and viability. The GO values simulated by GLMM Probit model were highly consistent with observed data (pseudo-*R*^2^ = 0.9925) (Supplementary Figure 4). The oospores reached the maximum germination at 111–125 days (DFO_50_), during grapevine dormancy (BBCH = 0), in MT samples, and at 131–165 days (DFO_50_), end of dormancy, in MTc samples (Supplementary Table 2 and Figure 3). Bootstrap analysis showed that DFO_50_ estimates for MT samples (Mean DFO_50_ = 117; LL_95_ = 112; UL_95_ = 122 days) were significantly lower than those obtained for MTc samples (Mean DFO_50_ = 151; LL_95_ = 138; UL_95_ = 164 days). Following trypan blue staining, the MTc oospores showed either an uncolored structure (Figures 4A–C), with a regular central ooplast surrounded by small-sized lipid globules typical of a mature spore (Vercesi et al., 1999), or an altered structure associated with the absence of the ooplast, coalescent lipid bodies, and a blue coloration indicating the loss of structural integrity (Figures 4D–F). During overwintering (Figure 4G), the percentage of VO significantly increased (*F* = 16.6; df = 3–32; *p* < 0.0001) from 9% (0 DFO; BBCH = 0), first to 17% (60 DFO; BBCH = 0) and then to 30% (120 DFO; end of grapevine dormancy, BBCH = 0). No further significant increases (VO = 37%) occurred at 180 DFO (grapevine sprouting, BBCH = 19) (Figure 4G), as occurring with G values (Figure 4G and Table 1).

### Estimation of the Oospore Post-maturation Period

**FIGURE 5:**
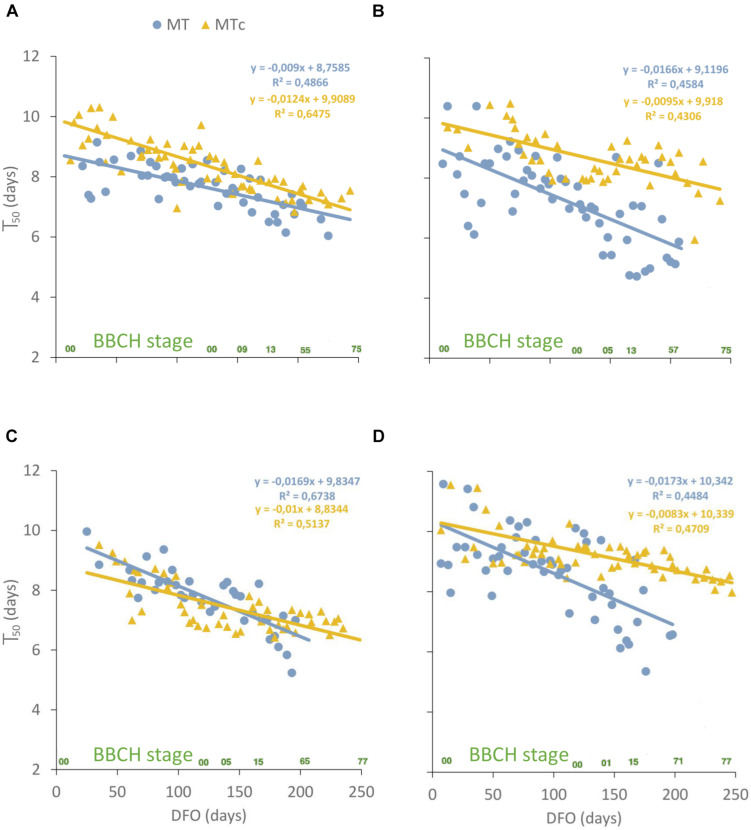
Scatterplot showing the T_50_ values, measured in days, at different days from the start of overwintering (DFO) for MT (blue dots) and MTc (yellow triangles) samples during year 1 **(A)**, 2 **(B)**, 3 **(C)**, and 4 **(D)**. Linear regression parameters and *R*^2^ are highlighted in blue for MT and yellow for MTc samples. The phenological stages of grapevine (BBCH scale) are reported in green on the *x*-axis.

GLM described satisfactorily Gcum over dai (pseudo-*R*^2^ = 0.92 ± 0.02 SD) and allowed to calculate the time to reach the maximum germination (T_50_) at germination assays with G > 0. Significant differences were found among T_50_ values at different DFO intervals in all sample series (Table 2). In particular, the T_50_ values of MT samples significantly reduced from 8–10 dai at 0–150 DFO interval (BBCH = 0, grapevine dormancy) to 6–7 dai at 151–240 DFO (BBCH = 1–79, from sprouting to fruit development). On the contrary, in MTc samples, significant reductions in T_50_ values from 9–10 to 7–8 dai could be observed already at grapevine dormancy (91–150 DFO), during years 2 and 3, and at late developmental stages (BBCH = 11–79, stages going from leaf to fruit development) during years 1 and 4. Linear correlation was performed between the T_50_ values of MT and MTc oospores and the phenological stages of grapevine at different DFOs. T_50_ values reduced over time following a significant (*p* < 0.0001), negative linear regression in both MT and MTc sample series (Figure 5). The comparison of the regression lines of MT and MTc within a year showed that either the slopes of the two sample series were identical but with different elevations, as observed in year 1 (*p* = 0.07 for the slope; *p* < 0.0001 for the elevation), where the regression line of MTc is higher than that of MT samples (Figure 5A), or the slopes were significantly different, as observed during years 2 (*p* = 0.03), 3 (*p* = 0.007), and 4 (*p* = 0.0008) (Figures 5B–D). The decrease of T_50_ values over DFO of MTc samples between years 2 and 4 was slower than that of MT samples, as demonstrated by the values of the slopes reported in Figures 5B–D.

**TABLE 2:** Phenological stages of grapevine (BBCH scale^*a*^) and average T_50_ values (measured in days) recorded each year by the oospores overwintered in natural (MT) and controlled conditions (MTc) at each DFO interval and results of statistical analysis (ANOVA and multiple comparison of mean values^*b*^).

DFO	Year 1			Year 2			Year 3			Year 4		
				
	BBCH	T_50_		BBCH	T_50_		BBCH	T_50_		BBCH	T_50_	
								
		MT	MTc		MT	MTc		MT	MTc		MT	MTc
0–30	0	7.7 ab	9.9 a	0	8.6 a	9.7 a	0	10 a	–	0	9.4 a	10.8 a
31–60	0	8.4 a	9.3 a	0	8 a	9.8 a	0	9.3 ab	9.2 a	0	9.5 a	10 ab
61–90	0	8.2 a	8.9 ab	0	8.2 ab	9.4 a	0	8.6 ab	8.6 a	0	9.6 a	9.3 bc
91–120	0	8 ab	8.6 ab	0	7.8 ab	8.5 abc	0	8.2 bc	7.5 b	0	8.7 a	9.2 bc
121–150	0	7.7 ab	8.2 abc	0	6.7 bc	8 bc	0	8.1 bc	6.8 c	0	8.9 a	9.2 bc
151–180	1–9	7.4 bc	7.8 bc	1–13	6.2 c	8.7 abc	1–13	7.3 cd	7 c	1–19	7.1 b	9.2 bc
181–210	11–61	6.8 c	7.4 c	14–57	6 c	8.5 abc	15–65	6.5 d	7 c	53–69	6.6 b	8.9 bc
211–240	63–75	6.3 c	7.3 c	60–75	6.5 bc	7.5 c	68–77	–	6.9 c	71–79	6.6 b	8.4 c
*F*		8.9	14.4		6.4	8.6		8.3	24.9		7.90	7.1
df		7.40	7.51		7.47	7.36		6.39	6.39		7.42	7.46
*p*		<0.001	<0.001		<0.001	<0.001		<0.001	<0.001		<0.001	<0.001

*Different letters correspond to significant differences for α = 0.05. ^*a*^0 = dormancy; 1–8 = sprouting; 11–19 = leaf development; 53–57 = inflorescences emerge; 60–68 = flowering; 71–79 = development of fruits. ^*b*^Different letters correspond to significant differences within a year for α = 0.05.*

## Discussion

In this study, the existence of synchrony between the pathogen (*P. viticola*) and the host plant (grapevine) has been investigated by comparing the phenology of oospore subpopulations overwintered in the same conditions of grapevine (MT samples) or in controlled conditions in laboratory (MTc samples). We followed the hypothesis that, if any connection with grapevine phenology existed, the phenology of MT samples, but not MTc samples, would follow grapevine phenology. To this purpose, we first identified the main factors influencing oospore germinability and then estimated and compared two important stages of oospore phenology, maturation (DFO_50_) and post-maturation (T_50_), of MT and MTc samples at different phenological stages of grapevine going from dormancy to fruit set.

### Cumulated Temperatures and Time From the Start of Overwintering Mainly Influence the Oospore Germinability

Average temperatures and rainfall occurring in vineyard 40–60 days before sampling are known to influence the oospore germination in laboratory (Vercesi et al., 2000). In this work, we further analyzed these parameters (as T, SOT, R, FR, and CR) and added the effect of time (as DFO) to identify which are the most important factors acting on oospore germination in isothermal conditions and use them for describing the maturation processes. PCA showed that, together, the investigated parameters explained 76% of the total variance, indicating that the most important meteorological factors have been considered. However, we cannot exclude the fact that other factors such as soil humidity, not investigated in the present study, could influence the oospore germination. The most important factors, all clustering together and negatively correlated with G, were T, SOT, CR, and DFO. All these variables are influenced by time, since average temperatures change with seasonality and cumulated temperatures and rainfall increase with time. This could indicate that time has a strong influence on oospore germinability. Decreasing germination rates were observed as DFO values increased, as previously reported in the literature for the oomycete *P. viticola* (Burruano et al., 1987; Ronzon-Tran Manh Sung and Clerjeau, 1988). A lower germinability with increasing spore age has also been observed in fungal species belonging to *Fungi* (Lücking et al., 2021) such as *Penicillium chrysogenum* (Ehgartner et al., 2016), *Aspergillus fumigatus*, *A. niger*, *Neurospora crassa* (Dantigny and Nanguy, 2009), and ascospores of *Talaromyces macrosporus* (Dijksterhuis and Teunissen, 2003). The negative correlation between G, T, and SOT indicates that the progressive increase of average temperatures recorded in vineyard is negatively affecting the oospore germination. Indeed, the maximum temperatures in late spring–summer often overcome 32°C, a value inhibiting oospore germination (Laviola et al., 1986), leading to a reduction in germinability. The results obtained on the rainfall variables, showing a higher correlation with CR than with R and FR, could indicate that oospore germinability is more influenced by the progressive increase, than by the entity and frequency of rainfall. The reduced influence of R and FR could also indicate that the oospores overwintered in presence of an adequate soil moisture in field. Soil moisture is a determinant factor for oospore germination (Burruano et al., 1992; Rossi and Caffi, 2007), and the absence of a water supply during overwintering rapidly leads to the absence of germination in the oospore population (Vercesi et al., 2010). In future studies, soil moisture should be deeply investigated with *ad hoc* sensors in vineyard. The separation of the germination percentage of the oospores (G), the sum of temperatures (SOT), and the number of days passed from overwintering (DFO) from the other variables over the second component suggested that these parameters had a very important influence on the oospore germination.

### Oospore Maturation in Field Is Completed During Grapevine Dormancy

Overall, the maturation process was completed at different timings in the two sample series. MTc samples showed higher values of DFO_50_ than MT samples. This difference could be due to the different storage conditions: while in controlled conditions the oospores were kept at constant regimes of temperature and water, in field conditions, the samples were exposed to fluctuating parameters that probably led to a faster maturation of the oospores. Notably, the faster maturation corresponded to a lower germination percentage. An earlier maturation in natural conditions, associated with reduced germination rates compared to artificial overwintering, has already been reported in the literature (Ronzon-Tran Manh Sung and Clerjeau, 1988). In general, the oospores of MT samples completed the maturation period, reaching a stable structure and the highest germination percentages, at about 120 DFO, before grapevine sprouting (that occurred at 151–180 DFO). On the contrary, MTc samples reached maturity at about 150 DFO, close to grapevine sprouting. These results indicate that the process of maturation is indeed completed in winter, as previously described (Vercesi et al., 1999), but with an increasing trend that allows the oospores to reach the highest percentage of viability and germination when grapevine approached the end of the dormancy state. Interestingly, the oospores overwintered in the field (MT) showed the same G values as those overwintered in controlled conditions (MTc) until 120 DFO (grapevine dormancy). Then, the two sample series started diverging: MTc samples not only showed higher germination rates, but also kept their germinability until the end of the experimental activities. In this case, at increasing age of the spores, no reduction in G occurred, suggesting that the favorable storage conditions (5°C and constant water availability) prolonged the oospore germinability. By contrast, MT samples showed a decreasing trend that led to a sporadic or absent germination at 211–240 DFO (phenological stage of grapevine going from flowering to fruit development). These results confirm previously data where a decrease in the germination of field samples (Ronzon-Tran Manh Sung and Clerjeau, 1988; Burruano et al., 1989; Vercesi et al., 2010) and an increased and prolonged germination of samples stored in cold conditions (3–12°C) (Burruano et al., 1990; Vercesi et al., 2010) have been observed. It must be pointed out that the decrease in germination observed in MT samples after the completion of the maturation period is not necessarily related with a loss of viability, but could be more likely due to seasonal variation leading to unfavorable conditions for germination (e.g., excessive temperatures) (Laviola et al., 1986). It is known, in fact, that the oospores can survive for more than a growing season (Kennelly et al., 2007) and previous tests showed that oospore viability in field samples is maintained in summer, even in absence of germination (Toffolatti et al., 2007).

The results obtained by trypan blue staining confirmed that the cytological changes associated with maturation were completed by the end of grapevine dormancy also in controlled conditions (MTc samples). During the maturation period, several modifications occur to allow the oospore to reach a stable structure, able to survive unfavorable weather conditions. Mature oospores possess a thick wall, a central ooplast, and small lipid globules at the periphery of the spore (Vercesi et al., 1999; Burruano, 2000). All these structures could be observed in the unstained oospores of this study. In the blue-colored oospores, the cytological changes associated with maturation did not occur regularly, confirming that part of the oospore population does not accomplish the maturation process and degenerates during overwintering (Vercesi et al., 1999). Previous studies showed that the percentage of VO increases from November to April (Tran Manh Sung et al., 1990; Burruano, 2000). The results obtained in the present study on oospore viability confirmed these results, showing that, due to the accomplishment of the maturation process, viability increased up to 40% during dormancy, with no further significant increase from 120 to 180 DFO, and that most of the oospores (about 60%) degenerated during the maturation process.

### Post-maturation Length Is Shorter at Grapevine Sprouting in Field Samples

The length of the post-maturation period was determined at all germination assays in order to investigate if this interval of time is equal or if it changes with time and phenological stages of grapevine in both overwintering conditions. Logistic regression of the cumulative G values at each germination assays fitted the data well and allowed us to calculate T_50_ values for almost all MT and MTc samples, excluding the tests where no germination occurred. In previous studies, the post-maturation period length at 20°C ranged between 7 and 9 days (Ronzon Tran Manh Sung and Clerjeau, 1989). Analogous values were observed in the present work at all DFO intervals for MTc samples and until the end of grapevine dormancy in MT samples. Starting from grapevine sprouting, T_50_ values of MT oospores decreased to 6–7 days. In general, the post-maturation period was longer in artificial than in field conditions, but in both cases, T_50_ showed a significant decreasing linear correlation with the time spent from overwintering, more pronounced in the case of the field conditions treatment. In MT samples, indeed, a significant decrease in the T_50_ values occurred passing from grapevine dormancy (0–150 DFO) to bud burst and leaf development (151–180 DFO), when the plant tissues became receptive to the pathogen (Gessler et al., 2011). In the following phenological stages of grapevine, going from inflorescence emergence to fruit development, this parameter did not further decrease. It must be pointed out that from 211 DFO onward, the germinability of oospores kept in the field was quite sporadic, as previously reported. The different post-maturation length of MT and MTc oospores and the decrease of T_50_ in MT samples at the start of grapevine growing season indicated the presence of synchrony of the pathogen with the host in field. A relationship between phenology of parasites and animal hosts has been previously observed in cases where there is a preferential parasitism of a particular host development stage (Dick, 1992; Hajek et al., 2018) and was confirmed also in the case of plant hosts. In the oak-powdery mildew pathosystem (Desprez-Loustau et al., 2010), both sporulation by the pathogen and the host susceptible stage were delayed with increasing altitude of the sites, and in the blueberry–*Monilinia vaccinii-corymbosi* pathosystem, germination of the overwintering structures synchronized with bud break of the host (Lehman and Oudemans, 2000). In the present study, it is clear that MT showed a different trend from MTc samples in T_50_ values, which significantly decreased when the plant reached susceptibility to the pathogen: at BBCH = 13 (leaf development stage), in fact, the leaves are flat and the stomata can be infected by the zoospores produced in the macrosporangia formed by the germinating oospores. A reduced interval of time in the oospore latency period has been indeed observed when primary infections occur (Pertot and Zulini, 2003). The post-maturation length in MTc samples was less related to the phenological stages of grapevine, since significant reductions in T_50_ values were observed already during grapevine overwintering. However, the occurrence of a significant, negative, linear correlation between T_50_ and DFO in MTc samples, even if with a milder slope than that of MT samples, could indicate that the oospores maintained a tendency toward a more rapid production of inoculum even when they did not perceive environmental changes. This suggests that, apart from exogenous factors, unknown endogenous factors could be regulating the oospore germination, as already observed in samples overwintered in controlled and natural conditions (Vercesi et al., 2010).

## Conclusion

In conclusion, the results obtained in this study confirmed the important role of temperatures (as average and cumulated values) and rainfall (as cumulated values) on the germination of the oospores overwintering in the open field. Furthermore, in the presence of adequate temperature and rainfall conditions, the most indicative parameters for analyzing the oospore germinability were the cumulated temperatures (SOT) and the time from the start of overwintering (DFO) parameters. The analysis of the oospore germination dynamics over four consecutive years highlighted that overwintering conditions influenced both the germination rates and the timing of the maturation post-maturation processes with specific periods of grapevine phenology. In particular, in field overwintering, the oospores completed maturation before the end of grapevine dormancy, germinated more rapidly at grapevine sprouting, and significantly decreased the germination rate starting from full flowering onward. These parameters seemed to be less related to grapevine phenology in controlled conditions: in this case, the maturation process was completed almost at grapevine sprouting, the post-maturation period was longer and decreased more slowly, and the oospores continued to germinate at high rates at later phenological stages of grapevine. Overall, this study demonstrates the strong adaptation of the pathogen to its host and opens interesting new perspectives on research into the factors regulating synchrony of pathogen and plant phenology, such as common environmental conditions or plant signals perceived by the pathogen. It has been demonstrated, for example, that the germination of the resting structures (sclerotia) of the onion white rot agent is stimulated by volatile compounds emitted by the host roots (Coley-Smith, 1960). Further studies on the oospore germinability in field conditions are, moreover, needed to investigate if maturation and post-maturation values show any connection with the development of primary infections and can be exploited for disease forecasting. Off-season survival is, in fact, a key determinant of the epidemic in the next season (Tack and Laine, 2014), and the correlation between grapevine downy mildew incidence in fall, associated with oospore production, and the disease development in the following spring has already been described (Carisse, 2016). The development of a forecasting model, based on oospore germination and primary infection establishment, could help in identifying the right moment for the application of a fungicide treatment and achieving a better disease management. The identification of the right moment for the fungicide treatment is still difficult nowadays for what concerns primary infections, and this often leads to the application of unnecessary treatments that should be avoided.

## Data Availability Statement

The raw data supporting the conclusions of this article will be made available by the authors, without undue reservation.

## Author Contributions

GM wrote the manuscript. GR supervised statistical analyses and performed GLMM simulation. ST carried out all experimental activities, analyzed the data, and wrote the manuscript. All authors contributed to the article and approved the submitted version.

## Conflict of Interest

The authors declare that the research was conducted in the absence of any commercial or financial relationships that could be construed as a potential conflict of interest.
