# Supplementary material for: The Study of the Germination Dynamics of Plasmopara viticola Oospores Highlights the Presence of Phenotypic Synchrony With the Host
Source: Front Microbiol. 2021 Jul 8;12:698586. doi: 10.3389/fmicb.2021.698586 (PMC8297619; doi:10.3389/fmicb.2021.698586)
Supplement: Supplementary file 1 [file Data_Sheet_1.DOCX]

Supplementary Material

# Supplementary Appendix

**GLMM model analysis**

To determine the length of the maturation period, the oospore germination dynamics recorded by MT and MTc samples between years 1-4 were investigated by Generalized Linear Mixed Model (GLMM) defined as follows:

$$g\left[ p\left( Y_{\mathrm{ijk}}=y_{\mathrm{ijk}} \right) \right]=\eta_{\mathrm{ijk}}=\beta_{0}+\beta_{\mathrm{DFO}}x_{1ijk}+\beta_{j}+\beta_{SOT|k}x_{2ijk}+\varepsilon_{\mathrm{ijk}}$$

where p(Y_ijk_=y_ìjk_) is the cumulative proportion of germinated oospores and Y_ijk_ is the cumulative number of germinated oospores out of the total (*i.e.*: the number of germinated oospore out of the total counted after 20 ° C temperature exposure), observed at the 14^th^ dai for the i-th germination assay, the j-th experimental condition (laboratory or field), with j=(lab,field), and the k-th scenario, with k=(MTc1, MTc2, MTc3, MTc4, MT1, MT2, MT3, MT4 where the number represents the year); η_ijk_ is the linear predictor expressed as the inverse normal density function so that g[p(Y_ijk_=y_ìjk_)]=η_ijk_= Φ^-1^[p(y_ìjk_)], where g[.]=Φ^-1^[.] is the Probit link function (Faraway, 2006). Parameter β_0_=b_0_+u_0jk_ is the intercept, where b_0_ is the intercept’s fixed term and u_0jk_ is the intercept random component which represents the effect arising from the random combination of year and sample occurring in each k-th scenario; β_DFO_x_1ijk_ is time fixed effect where β_DFO_ is the fixed slope and x_1ijk_ is time expressed as DFO for the i-th germination assay, the j-th experimental condition, and the k-th scenario; β_j_ is the fixed experimental condition’s effect, actually representing the laboratory effect, as the field factor’s level is the reference; β_SOT|k_x_2ijk_ is the random temperature effect where β_SOT|k_ is the random slope for the computed within-scenario SOT and x_2ijk_ is the computed SOT for the i-th oospore germination assay, the j-th experimental condition and the k-th scenario; finally, ε_ijk_ is the error term for the i-th oospore germination assay, the j-th experimental condition and the k-th scenario. The structural dispersion heterogeneity due to the correlation between mean and variance in binomial data (Stroup, 2015) is managed by applying the total number of assessed oospores as weighs, in order to obtain a weighted parameter estimation for the GLMM Probit model just described above. The GLMM goodness of fit was obtained by observed vs simulated linear regression in order to compute the pseudo-R^2^ (Piñeiro *et al.*, 2008). The model parameters were then used to compute the DFO_50|k_ estimates for each scenario as follows:

$$\mathrm{DFO}_{50|k}=\frac{\left( -\beta_{0}-\beta_{j}-\beta_{SOT|k}x_{2ijk} \right)}{\beta_{\mathrm{DFO}}}$$

where DFO_50|k_ is the scenario-dependent time in the season at which the oospores reach 50% germination, completing maturation, and x_2ijk_ is set as SOT_50_, that is, the computed SOT at which the observed cumulative oospore germination is about 50%. The DFO_50|k_ estimates were then submitted to bootstrap algorithm in order to estimate the DFO_50|k_ expected value and 95% confidence limits for field and laboratory conditions (Efron & Tibshirani, 1986). These latter values are computed by performing 250 bootstrap replications and allow to individuate the DFO_50|k_ ranges (UL=upper limit; LL=lower limit) and compare the oospore population responses in controlled and field condition (Badiru & Ijaduola, 2009). GLMM Probit model is fitted by glmer() function implemented in lme4 R 3.4.3 package, whilst bootstrap computations are performed by bootstrap function implemented in bootstrap R 3.4.3 package.

*References*

Badiru AB, Ijaduola AO, 2009. Half-life theory of learning curves. *IEEE Systems Journal* **3**, 154–165.

Cox D, Snell E, 1989. *Analysis of binary data*. Boca Raton: Chapman and Hall/CRC.

Efron B, Tibshirani R, 1986. Bootstrap methods for standard errors, confidence intervals, and other measures of statistical accuracy. *Statistical Science* **1**, 54–75.

Faraway J, 2006. *Extending the linear Model with R*. Boca Raton, London, New York: CHAPMAN & HALL / CRC.

Piñeiro G, Perelman S, Guerschman JP, Paruelo JM, 2008. How to evaluate models: Observed vs. predicted or predicted vs. observed? *Ecological Modelling* **216**, 316–322.

Stroup WW, 2015. Rethinking the analysis of non-normal data in plant and soil science. *Agronomy Journal* **107**, 811–827.

Zar JH, 2010. *Biostatistical analysis*. Saddle River, New Jersey (USA): Pearson Prentice Hall.
