# Supplementary material for: The Study of the Germination Dynamics of Plasmopara viticola Oospores Highlights the Presence of Phenotypic Synchrony With the Host
Source: Front Microbiol. 2021 Jul 8;12:698586. doi: 10.3389/fmicb.2021.698586 (PMC8297619; doi:10.3389/fmicb.2021.698586)
Supplement: Supplementary file 2 [file Data_Sheet_2.docx]

Supplementary Material

# Supplementary Tables

**Supplementary Table 1**. Phenological stage of grapevine (BBCH scale^a^) and monthly values of average temperatures (T), sum of temperatures (SOT), total rainfall (R), cumulated rainfall (CR) and frequency of rainfall (FR) at different DFO intervals between years 1-4 in MT vineyard. Temperatures are expressed in °C, rainfall in mm.

| DFO | BBCH stage | Year | T | SOT | R | CR | FR |
| --- | --- | --- | --- | --- | --- | --- | --- |
| 1-30 | 0 | 1 | 10.2 | 305.3 | 42.4 | 42.4 | 18 |
| 31-60 | 0 | 1 | 5.7 | 477.5 | 52.2 | 94.6 | 13 |
| 61-90 | 0 | 1 | 2.7 | 558.3 | 48.8 | 143.4 | 9 |
| 91-120 | 0 | 1 | 2.1 | 620.6 | 0.0 | 143.4 | 0 |
| 121-150 | 0 | 1 | 8.5 | 875.6 | 1.8 | 145.2 | 3 |
| 151-181 | 1-9 | 1 | 12.1 | 1238.7 | 60.0 | 205.2 | 9 |
| 181-210 | 11-61 | 1 | 20.9 | 1865.3 | 12.9 | 218.1 | 4 |
| 211-240 | 63-75 | 1 | 27.7 | 2620.1 | 65.2 | 283.3 | 6 |
| 1-30 | 0 | 2 | 8.8 | 264.2 | 113.0 | 113.0 | 8 |
| 31-60 | 0 | 2 | 4.3 | 392.0 | 70.0 | 183.0 | 8 |
| 61-90 | 0 | 2 | 2.0 | 451.2 | 29.0 | 212.0 | 5 |
| 91-120 | 0 | 2 | 3.7 | 561.3 | 86.0 | 298.0 | 9 |
| 121-150 | 0 | 2 | 7.8 | 795.8 | 76.0 | 374.0 | 7 |
| 151-181 | 1-13 | 2 | 12.7 | 1175.9 | 96.0 | 470.0 | 12 |
| 181-210 | 14-57 | 2 | 15.9 | 1653.0 | 84.0 | 554.0 | 11 |
| 211-240 | 60-75 | 2 | 20.1 | 2157.7 | 56.0 | 610.0 | 8 |
| 1-30 | 0 | 3 | 8.8 | 208.1 | 114.0 | 114.0 | 16 |
| 31-60 | 0 | 3 | 5.3 | 348.0 | 55.0 | 169.0 | 10 |
| 61-90 | 0 | 3 | 2.2 | 389.0 | 18.4 | 187.4 | 12 |
| 91-120 | 0 | 3 | 2.1 | 440.8 | 11.6 | 199.0 | 6 |
| 121-150 | 0 | 3 | 6.7 | 723.5 | 39.8 | 238.8 | 7 |
| 151-181 | 1-13 | 3 | 11.4 | 1099.5 | 112.4 | 351.2 | 13 |
| 181-210 | 15-65 | 3 | 17.2 | 1641.0 | 78.0 | 429.2 | 11 |
| 211-240 | 68-77 | 3 | 20.0 | 2256.1 | 56.7 | 485.9 | 5 |
| 1-30 | 0 | 4 | 7.4 | 111.6 | 0.0 | 0.0 | 0 |
| 31-60 | 0 | 4 | 2.8 | 150.6 | 1.4 | 1.4 | 2 |
| 61-90 | 0 | 4 | 0.5 | 199.8 | 9.0 | 10.4 | 7 |
| 91-120 | 0 | 4 | 3.9 | 350.9 | 61.6 | 72.0 | 12 |
| 121-150 | 0 | 4 | 6.5 | 639.2 | 41.0 | 113.0 | 9 |
| 151-181 | 1-19 | 4 | 12.6 | 1097.4 | 78.4 | 191.4 | 13 |
| 181-210 | 53-69 | 4 | 16.9 | 1618.8 | 61.2 | 252.6 | 9 |
| 211-240 | 71-79 | 4 | 17.6 | 2345.0 | 23.0 | 275.6 | 3 |

^a^0=dormancy; 1-8= sprouting; 11-19=leaf development; 53-57=inflorescences emerge; 60-68=flowering; 71-79=development of fruits.

**Supplementary Table 2.** GLMM parameters, *i.e.* model’s intercept (β_0_= b_0_+u_0k_), DFO’s fixed slope (β_DFO_), experimental conditions’ effect (β_j_), SOT’s within-scenario random slopes (β_SOT|k_) and fixed parameter’s p-values p(z), and within-scenario computed SOT_50_ (°C) and DFO_50_ (days).

| Scenario (k) | b_0_ | u_0jk_ | β_0_ | β_DFO_ | β_j_ | β_SOT\|k_ | SOT_50_ | DFO_50_ |
| --- | --- | --- | --- | --- | --- | --- | --- | --- |
| MTc1 | -2.477,00 | 0.2 | -2.3 | 0.214, | -0.6075, | -0.0004 | 730 | 147 |
| MTc2 | p(z) < 0.001 | 0.2 | -2.3 | p(z) < 0.001 | p(z) < 0.001 | 0.0002 | 659 | 131 |
| MTc3 |  | -0.1 | -2.6 |  |  | -0.0003 | 750 | 158 |
| MTc4 |  | 0.4 | -2.1 |  |  | -0.0010 | 860 | 165 |
| MT1 |  | 0.0 | -2.4 |  |  | -0.0001 | 590 | 117 |
| MT2 |  | -0.3 | -2.8 |  |  | 0.0006 | 529 | 115 |
| MT3 |  | -0.6 | -3.0 |  |  | 0.0007 | 547 | 125 |
| MT4 |  | -0.1 | -2.6 |  |  | 0.0009 | 271 | 111 |
