# Supplementary material for: The Study of the Germination Dynamics of Plasmopara viticola Oospores Highlights the Presence of Phenotypic Synchrony With the Host
Source: Front Microbiol. 2021 Jul 8;12:698586. doi: 10.3389/fmicb.2021.698586 (PMC8297619; doi:10.3389/fmicb.2021.698586)

Supplementary Material

## Supplementary Figures

**Supplementary Figure 1.** Preparation of the oospore samples for the germination assays. Leaves showing downy mildew symptoms were collected from the field and observed at the dissecting microscope to cut fragments (1 cm diameter) rich in oospores (A). Nylon bags, each one containing 20 leaf fragments were prepared (B) and divided into two groups (C): the first one, consisting of three series of 70 bags, was overwintered close to the plants on the soil surface using anti-hail nets and weights to prevent the loss of the samples (C); the second group, consisting of three series of 70 bags, was overwintered in laboratory conditions, at 5 °C and constant humidity (D). The three series of samples were overwintered in different places inside the vineyard and in three different incubators in laboratory. Germination assays were carried out twice a week for 35 weeks (corresponding to different phenological stages of grapevine, in green) for both overwintering conditions (E).


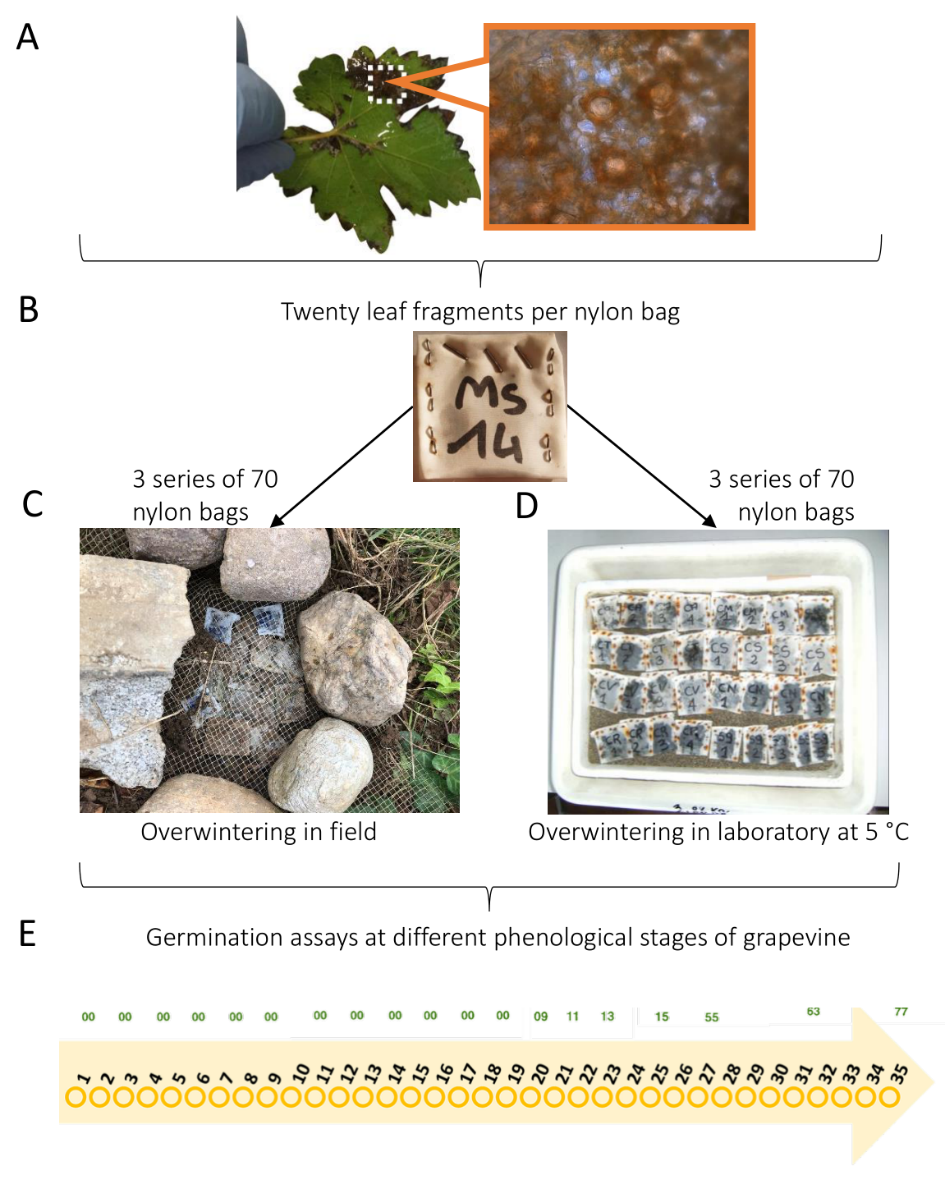


**Supplementary Figure 2.** Scheme of the germination assays. The three nylon bags prepared for each germination assay were collected from the overwintering site (A). The oospores were separated from the leaf tissues through homogenization in glass Potters, filtered to remove undesired leaf debris and resuspended in water (B). Four droplets of 10 µL containing 100 oospores were plated on three Petri dishes (6 cm diameter) containing 1% water-agar and the number of germinated oospores was daily counted from 1 to 14 dai (day from the start of incubation) at 20 °C (C).


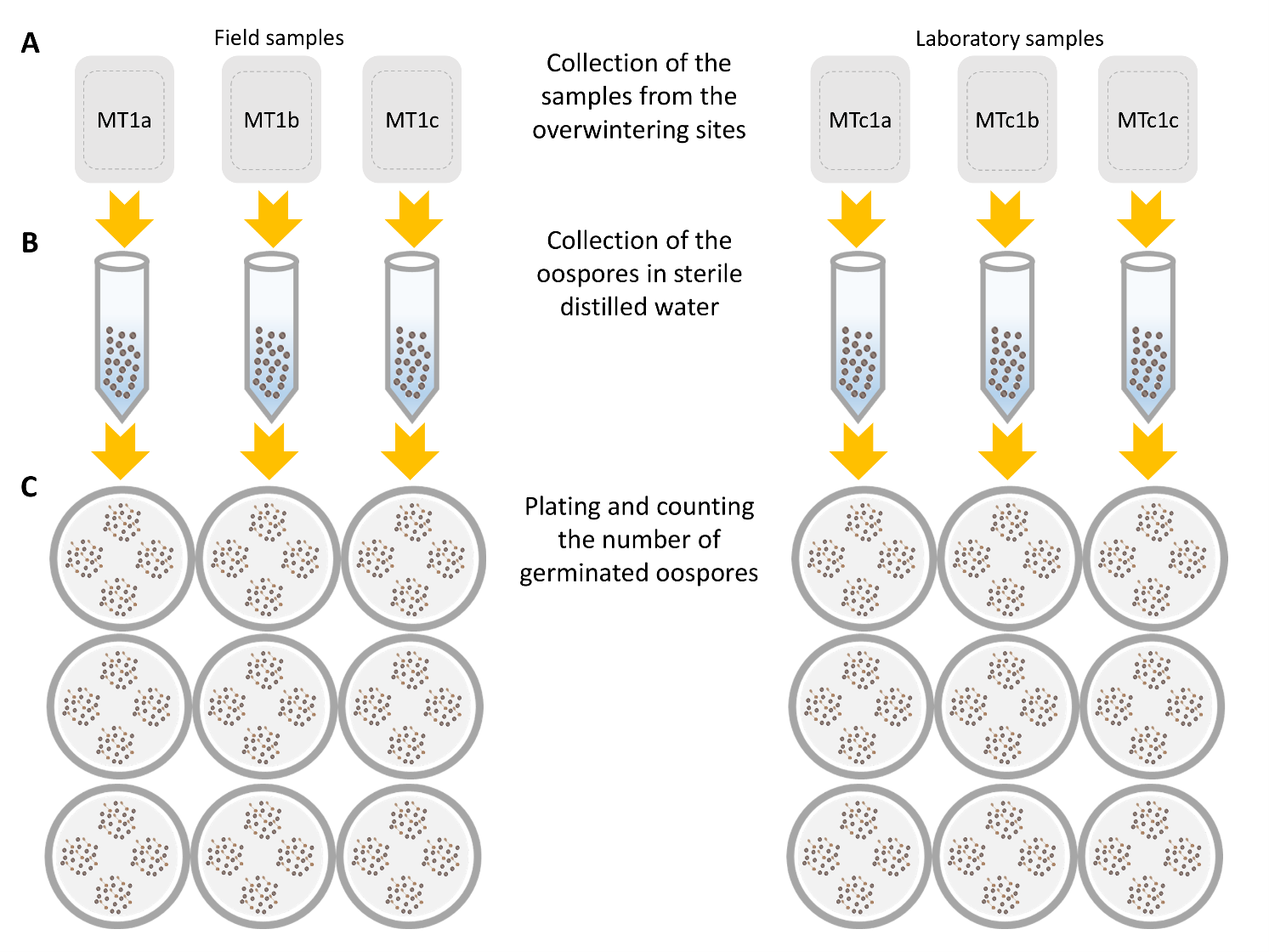


**Supplementary Figure 3.** Examples of datasets used to estimate the post-maturation (A) and maturation parameters (B). A) Cumulated number of germinated oospores (Gcum) at each dai recorded in the germination assay carried out at 123 DFO in MT samples of year 2. B) Cumulated values of Gcum (GO) recorded at 14 dai in each germinaton assay over days from overwintering (DFO) for MT samples in year 3. Vertical bars represent standard deviation.


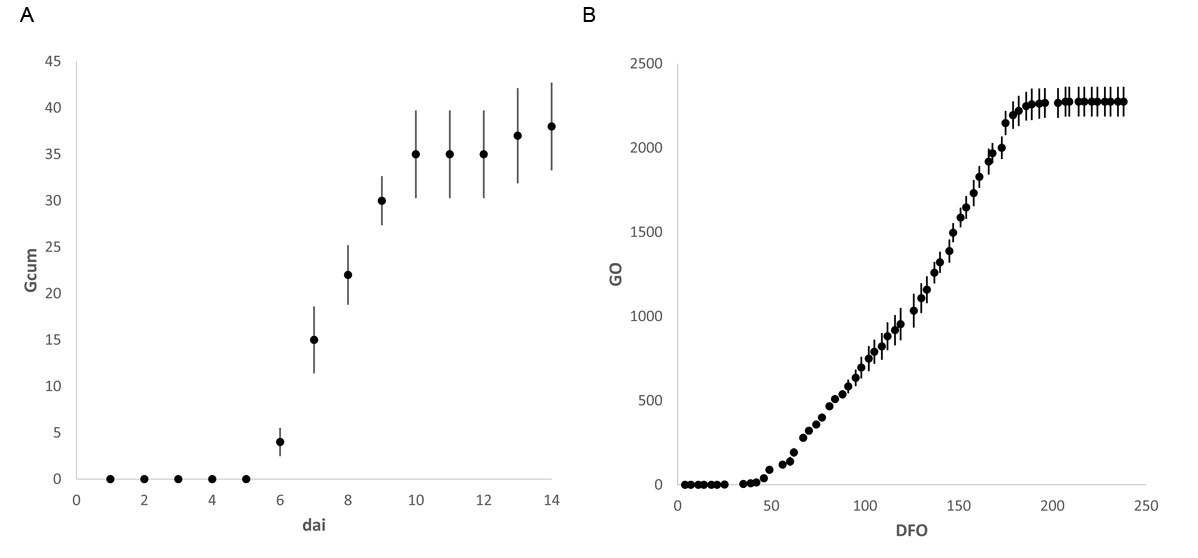


**Supplementary Figure 4.** GLMM Probit model goodness of fit.


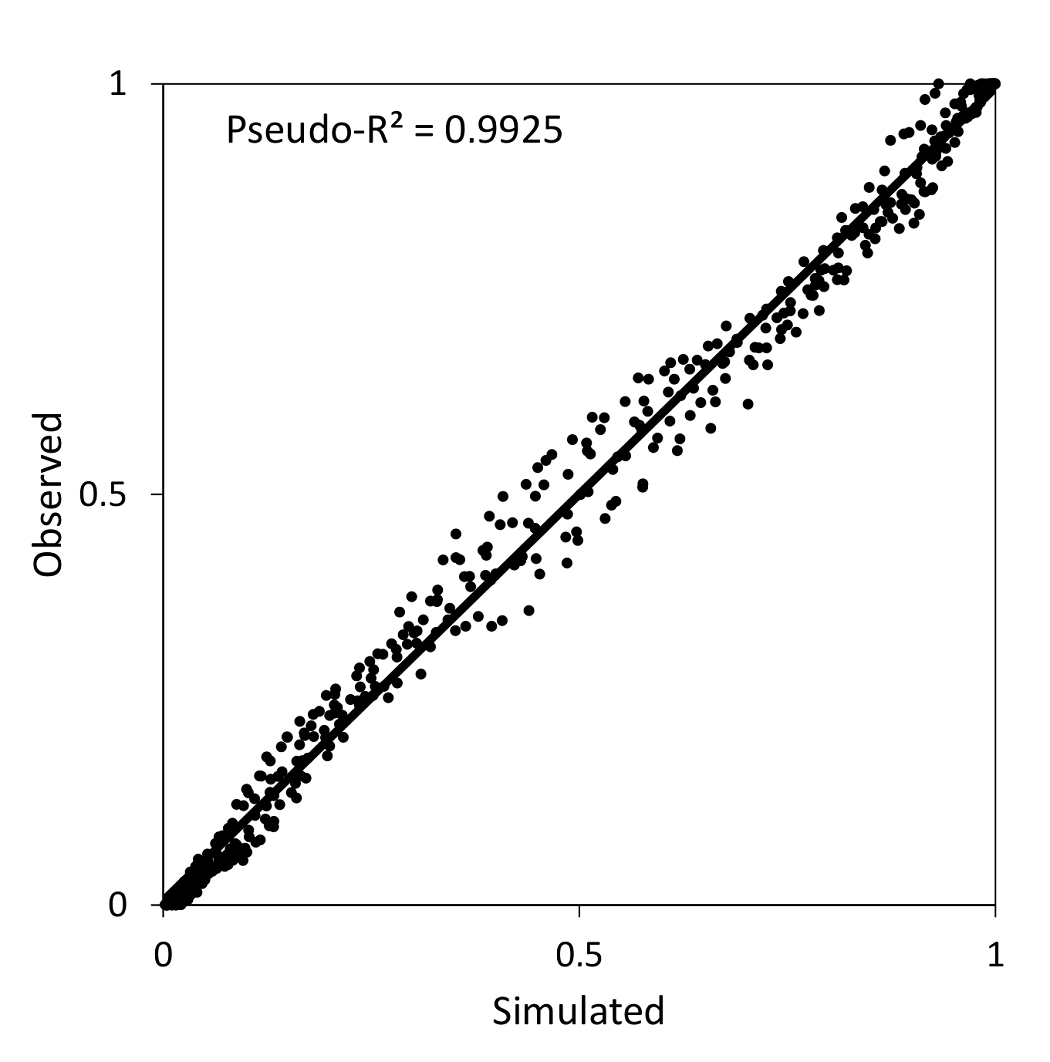

Supplement: Supplementary file 3 [file Data_Sheet_3.docx]
